# Clinicopathology and Recurrence Analysis of 44 Jaw Aneurysmal Bone Cyst Cases: A Literature Review

**DOI:** 10.3389/fsurg.2021.678696

**Published:** 2021-06-23

**Authors:** Yu Liu, Jinhan Zhou, Jue Shi

**Affiliations:** Key Laboratory of Oral Biomedical Research of Zhejiang Province, School of Stomatology, The Affiliated Hospital of Stomatology, Zhejiang University School of Medicine, Hangzhou, China

**Keywords:** aneurysmal bone cyst, recurrence, clinicopathology, jaw bone, radiograph

## Abstract

In the past half-century, considerable attention has been paid to oral and maxillofacial skeletal cyst, however, aneurysmal bone cyst (ABC), unlike other common bone diseases, still contours numerous unanswered questions in terms of classification, etiology and pathological mechanism. The purpose of this article was to evaluate the proportion of primary ABC and secondary ABC, and to assess the recurrence of ABC and related factors. A methodical search of Embase, MEDLINE, Cochrane Library, Web of Science was conducted for well-documented jaw aneurysmal bone cyst (JABC) cases. One hundred thirty-one articles were identified after database searching and 31 of them were included in our study for further research with 44 JABC cases. All the articles were analyzed by two separate authors. About 25% of the reported jaw aneurysmal bone cyst was secondary. Both the pathological classification and surgical treatment had a significant influence on recurrence rate (*P* = 0.0082, *P* = 0.0022), while patients' age or radiographic features rarely affected prognosis. Jaw aneurysmal bone cysts can present variable clinical and histological presentations. Recurrence may be attributed to omittance of underlying potential blood supply or conservative surgical protocol.

## Introduction

Aneurysmal bone cyst (ABC) is a common skeletal bone lesion that usually observed in long bones and vertebra. Only 1.8% of ABCs, however, occur in the jaw bones (JABC). This rare occurrence was firstly recognized by Jaffe and Lichtenstein in 1942 (1). Several misconceptions still stand due to its scarceness and diversity in clinicopathology. In most cases, JABC presents itself as a rapid-growing, painful or painless, swelling defect with or without bony expansion (1). In some cases, it may be misdiagnosed as a malignant disease. Occasionally, however, an asymptomatic, slow-growing lesion can also be found as JABC. More recently, a review has updated the recurrence rate of head and neck ABCs with 94.4% patients free of disease in the average follow-up period of 3.59 years (2). This result was widely different from the old ones, which ranged from 21 to 50%. However, the risking factors of ABC recurrence remains an ongoing challenge due to the diverse clinical features and pathogeny.

For a very long time, ABC had been considered as a pseudocyst of hemodynamic or reaction origin, in other words, “primary ABC” or “secondary” to other diseases. Primary aneurysmal bone cyst could result from any traumatic cause which leads to intramedullary or subperiosteal hemorrhage if followed by an inaccurate repair process, or other hemodynamic states, it can create an enlarging vascular bed and erosion of bone (1, 3). The terminology “secondary aneurysmal bone cyst” is characterized as a haemorrhagic reaction of a pre-existing bone lesion. The initial lesion could be totally replaced by a secondary ABC or remains a part of it (4). This uncertainty increases the difficulty of distinguishing a pure ABC and an affiliated one, although histopathology is essential in the final diagnosis.

This review aims to evaluate the proportion of primary ABC and secondary ABC, and to assess the recurrence of ABC and related factors, which has certain clinical significance for the diagnosis of oral and maxillofacial skeletal cyst.

## Materials and Methods

### Search Strategy and Selection Criteria

Articles published in English journals from 1958 to October 2019 that reported ABCs in the jaws (maxilla or mandible) were included in the present study. Reported ABCs in animals, in locations other than jaw bones, in languages other than English and cases with disputed description or diagnosis of ABC were excluded.

We searched online databases (Embase, MEDLINE, Cochrane Library, Web of Science), electronic abstract databases and references in published articles.

We used the following keywords:

Jaw aneurysmal bone cyst OR aneurysmal bone cyst.
AND clinical OR radiograph OR pathology OR treatment OR surgery.
AND recurrence OR recurrence rate OR relapse rate.
AND case OR review OR analysis.

Two authors cross-checked the relevant cases and determined a final list of included articles.

### Data Synthesis and Analysis

The proportion of different clinical features were calculated in this study, including gender, location, and radiographic behavior. We made estimates of the mean and standard deviation (SD). Chi-square test/Fisher's exact test was applied to evaluate the relation of recurrence and clinicopathology features and *P* < 0.05 was considered statistically significant. All statistical analyses were performed with GraphPad Prism 8.0 (GraphPad Software Inc., San Diego, CA, USA).

## Results

### General Description

**Figure 1:**
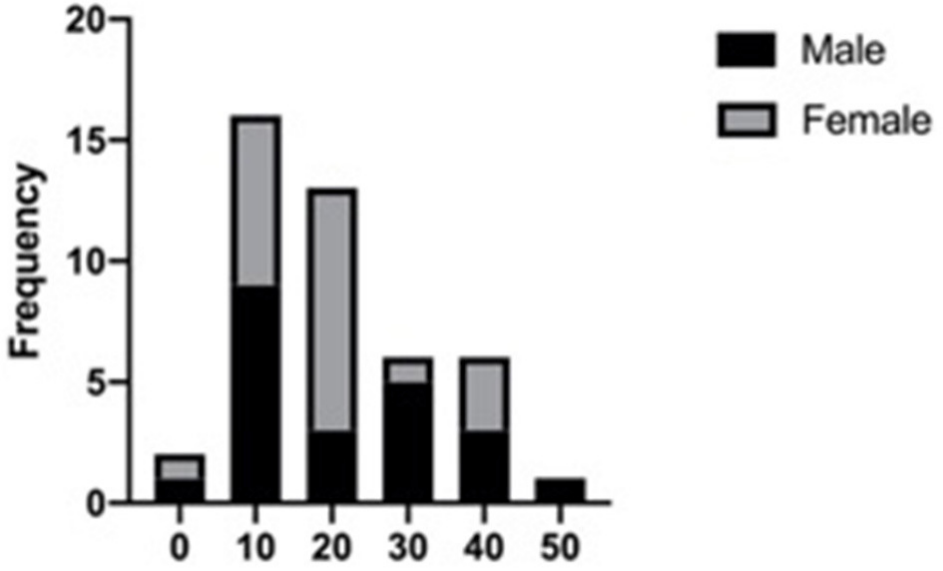
Age and gender groups.

**Figure 2:**
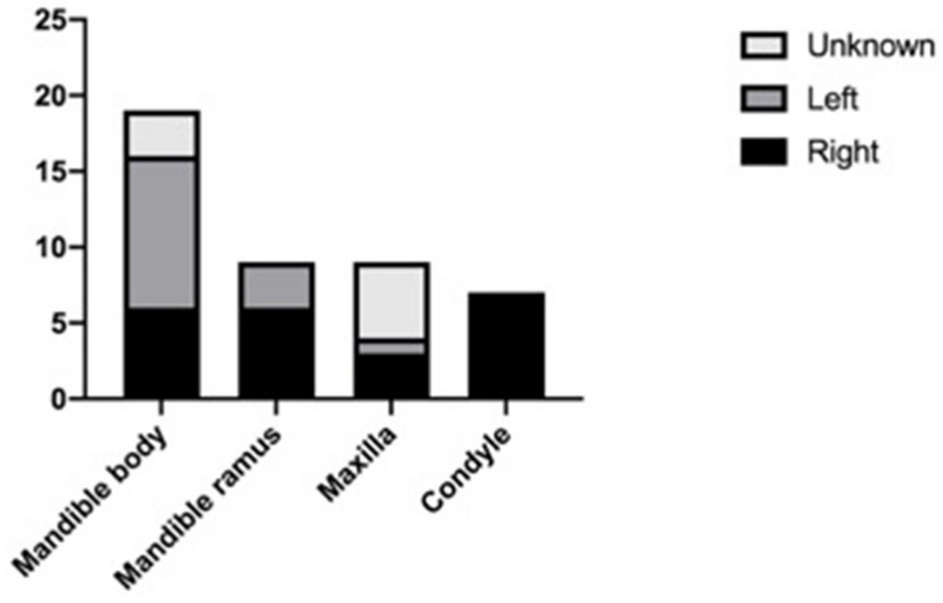
Location.

**Figure 3:**
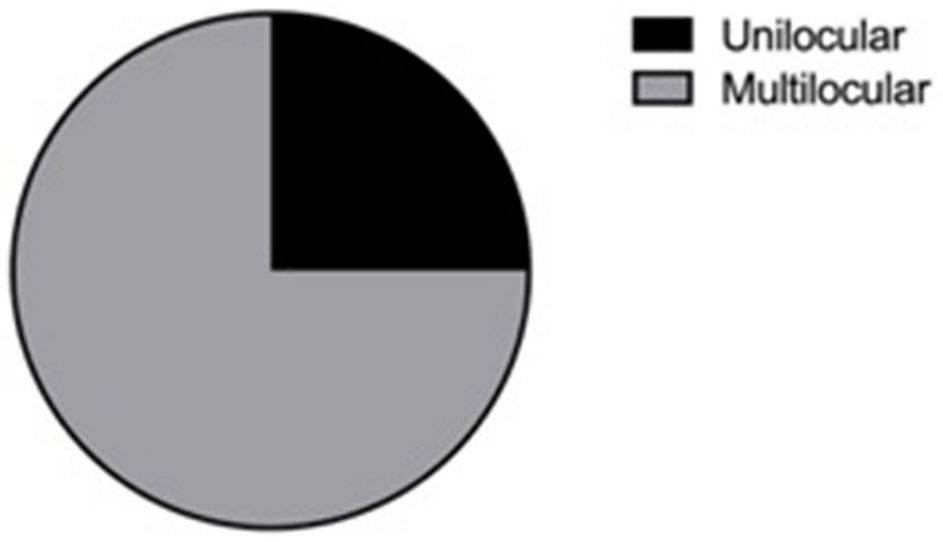
Locular.

After excluding articles as per criteria, 31 articles with 44 JABC cases were included in the present review, with 22 (50%) males and 22 (50%) females ranging in age from 3 to 48 years (mean age: 19.25 ± 15.46 years). There was no gender predilection. The age distribution, on the other hand, was significant, 63.64% (*n* = 28) of the lesions had occurred in the first 2 decades of life (*P* < 0.05). The distribution of cases according to age and gender groups is presented in Figure 1. Distribution of location were presented in Figures 2, 3, with 9 cases in maxilla (20.45%) and 35 cases in mandible (79.54%). Figure 3 showed that 75% of patients had multilocular presentation in radiographic examination and only 25% were presented as unilocular lesions. The majority of patients had shown with rapidly growing swelling as a significant clinical feature, in several cases also with localized pain. Table 1 encompasses data regarding the clinical features and other parameters of JABCs.

**Table 1:** Summary OF clinicoradiographic and histopathologic features of 31 studies of jaw aneurysmal bone cyst.

**St** **No**.	**References**	**Pt** **No**.	**Age**	**Gender**	**Location**	**Locular**	**History of** **trauma**	**Pathologic** **features**	**Surgery**	**Outcome** **/follow-up** **duration**	**Clinical** **features**
1	Wiatrak (5)	1	15	F	R Mand body	Multi	N	ABC only	Curettage	N, 2y	Swelling+Pain+Expansion
		2	16	F	L Mand body	Uni	N	ABC only	Curettage	N, 1.5y	Swelling+Expansion
		3	10	M	L Mand ramus	Uni	Y	ABC only	Hemi-mandibulectomy	N, 0.5y	Expansion
2	Pelo et al. (6)	4	10	M	R Mand condyle	Uni	N	ABC only	Low condylectomy	N, 1y	Swelling+Expansion
3	Zadik et al. (7)	5	37	F	R Mand condyle	Uni	N	ABC only	Curettage+marginal mandibulectomy	N, 5y	Swelling+Expansion
4	Verma et al. (8)	6	8	M	R Max	Multi	N	ABC only	Curettage	N, 3m	Swelling+Pain+Expansion
5	Debnath et al. (9)	7	8	M	R Max alveolar	Multi	Y	ABC only	Segimental maxillectomy	N, NA	Swelling+Pain+Expansion
6	Neuschl et al. (10)	8	28	M	L Mand ramus	Multi	N	ABC only	Segimental resection	N, NA	Swelling+Pain+Expansion
7	El Deeb et al. (11)	9	19	M	L Mand body	Multi	N	ABC with FD	Curettage	R, 0.5y	Swelling+Pain+Expansion +Paralysis
8	Zachariades et al. (12)	10	35	F	R Max	Uni	N	ABC only	Curettage	N, 2y	Swelling+Expansion
		11	37	M	R Mand ramus	Multi	N	ABC only	Curettage	N, 4y	Swelling+Expansion
9	Trent and Byl (13)	12	32	M	L Mand ramus	Multi	Y	ABC only	Curettage	R, 0.5y	Swelling+Expansion
									Partial left mandibulectomy	N, 2y	
10	Alvarez et al. (14)	13	27	M	L Mand body (chin)	Multi	Y	ABC only	Curettage	N, 2y	Swelling+Expansion
11	Saltzman and Jun (15)	14	22	F	L Mand body	Multi	NA	ABC only	Curettage+cryotherapy	N, 11y	Swelling+Expansion
12	Onerci and Ergin (16)	15	9	F	R Mand condyle	Multi	NA	ABC only	Condylectomy	N, 1.5y	Swelling+Expansion
13	Padwa et al. (17)	16	3	F	R Mand ramus	Multi	NA	ABC with OF	Curettage	R, 1.5y	Swelling+Expansion
									Curettage	N,1y	
		17	7	F	L Mand ramus	Multi	NA	ABC with FH	Partial left mandibulectomy	N, 2y	Swelling+Pain+Expansion +Paralysis
		18	5	F	L Mand body and ramus	Multi	NA	ABC with CGCG	Curettage Left hemi-mandibulectomy	R, 2m N, NA	Swelling+Expansion
14	Saheeb et al. (18)	19	13	M	L Mand body and ramus	Multi	N	ABC only	Curettage	N, 2y	Swelling+Expansion
		20	5	M	Primary palate	Multi	N	ABC with CF	Curettage	N, 2y	Swelling+Expansion
15	Marchetti et al. (19)	21	15	F	R Mand body and ramus	Uni	N	ABC only	Partial left mandibulectomy	N, 10y	Swelling+Pain+Expansion +Paralysis
		22	40	M	L Mand angle	Multi	NA	ABC only	Curettage Curettage	R, 8y N, 1y	Swelling+Expansion
		23	48	M	R Mand body	Multi	N	ABC only	Curettage	N, 5y	Swelling+Expansion
16	Toljanic et al. (20)	24	16	M	R Mand ramus	Multi	N	ABC only	Curettage Curettage+cryotherapy	R, 0.5y N,1y	Swelling+Pain+Expansion
17	Jacomacci et al. (21)	25	41	F	R Mand body	Uni	N	ABC with COD	Curettage	N, 1y	Swelling+Expansion
18	Chandolia et al. (22)	26	28	M	R Mand body	Uni	N	ABC with CGCG	Curettage	N, NA	Swelling+Expansion
19	Ziang et al. (23)	27	19	F	R Mand condyle	Multi	N	ABC only	Condylar resection	N, 0.5y	Swelling+Pain+Expansion
20	Motamedi (24)	28	18	M	R Mand condyle	Multi	N	ABC only	Curettage Curettage Marsupialization then curettage	R, 0.5y R, 0.5y N, 5y	Swelling+Expansion
21	Reddy et al. (25)	29	16	F	R Mand body	Multi	NA	ABC with OF	Hemi-mandibulectomy	N, 2y	Swelling+Expansion+Root resorption
22	Jeblaoui et al. (26)	30	24	F	R Mand ramus	Multi	NA	ABC only	Curettage	N, 4y	Swelling+Pain+Expansion
23	Breuer et al. (27)	31	6	M	R Mand ramus	Multi	NA	ABC only	Curettage	N, 2w	Swelling+Pain+Expansion
24	Peruma et al. (28)	32	12	F	L Mand body and ramus	Multi	NA	ABC only	Partial left mandibulectomy	N, 1.5y	Swelling+Expansion
25	Tabrizi et al. (29)	33	17	F	R Mand condyle	Multi	N	ABC with NOF	Condylar resection	N, 1y	Swelling+Pain+Expansion
26	Fyrmpas et al. (30)	34	12	F	L Max sinus	Uni	NA	ABC only	Curettage under endoscopy	N,9m	Swelling+Expansion
27	Ettl et al. (31)	35	17	F	R Mand condyle	Multi	NA	ABC only	Condylar resection	N, 8m	Swelling+Pain+Expansion
28	Sarode (32)	36	10	M	Max	Uni	NA	ABC with JPOF	Curettage Maxillectomy	R, 2y N, 3y	Swelling+Expansion
29	Gotmare et al. (33)	37	10	M	L Mand body	Multi	NA	ABC with JPOF	Curettage Hemi-mandibulectomy	R, 0.5y N, 6m	Swelling+Expansion
30	Gabric et al. (34)	38	11	F	L Mand body	Uni	N	ABC only	Curettage	N, 2y	Swelling+Expansion
31	Triantafillidou et al. (1)	39	7	F	Max sinus	Multi	NA	ABC with OF	Curettage Curettage Partial maxillectomy	R, NA R, NA N, 13y	Swelling+Expansion
		40	18	F	Max	Multi	NA	ABC only	Exicision	N, 8y	Swelling+Expansion
		41	21	M	Max sinus	Multi	NA	ABC only	Partial maxillectomy	N, 6y	Swelling
		42	35	M	Mand body	Multi	NA	ABC only	Curettage	N, 5y	Swelling+Expansion
		43	28	F	Mand body	Multi	NA	ABC only	Curettage	N, 4y	Swelling+Expansion
		44	32	M	Mand body	Multi	NA	ABC only	Exicision	N, 2y	Swelling

*St No., study number; Pt No., patient number; F, female; M, male; Max, maxillary; Mand, mandible; Uni, unilocular; Multi, multilocular; N, no history of trauma/recurrence; Y, had history of trauma; R, recurrence; NA, not available; FD, fibrous dysplasia; OF, ossifying fibroma; OH, fibrous histiocytoma; CGCG, center giant cell granuloma; CF, cementifying fibroma; COD, cemento-osseous dysplasia; NOF, nonossifying fibroma; JPOF, juvenile psammomatoid ossifying fibroma*.

### Recurrence Analysis

Focusing on the recurrence status, it was surprising to find a distinguishable division between primary ABC and secondary ABC, the latter possesses a higher recurrence rate than the former (*P* = 0.0082) (Table 2). A similar result was found when surgical procedure was involved (Table 3). Forty-four patients had experienced 55 surgery with 12 cases of recurrence. Patients underwent radical surgery such as Hemi-mandibulectomy and partial mandibulectomy retained a lower recurrence rate than conservative curettage or excision (*P* = 0.0022). Age and radiographic features (unilocular or multilocular) had no significant influence on the relapse rate.

**Table 2:** Relationship of pathology and recurrence rate (Chi-square test).

	**Recurrence**		***P*-value**	**Odds ratio**	**95%CI**
	**Yes**	**No**			
**Pathology**					
Primary ABC	4	28	0.0082	0.1429	0.03574–0.629
Secondary ABC	6	6			

**Table 3:** Relationship of surgical protocol and recurrence rate (Fisher's exact test).

	**Recurrence**		***P*-value**	**Odds ratio**	**95%CI**
	**Yes**	**No**			
**Surgical treatment**					
Conservative protocol	12	23	0.0022	Infinity	2.262-Infinity
Aggressive protocol	0	20			

## Discussion

Jaw aneurysmal bone cyst occurs predominately in young individuals under the age of 20 (3, 35). Most studies present a similar balance in gender difference (3, 4, 35). Our review showed similar results that are compatible with previously reported literature. Rutter et al. (36) and Biesecker et al. (37) found a slight bias toward female patients, as they accounted for 66 cases (59%) in a total sample of 105. Posterior of mandible is the most common site for JABC (35, 38). JABC can have variable clinical features, however, most of them would show swelling and bony expansions. In several cases, it also exhibits an asymptomatic lesion occasionally discovered as radiolucency on routine radiography. JABC has variable radiological appearances and should be considered in the differential diagnosis of any unilocular or multilocular radiolucency of the jaws and any mixed radiopaque-radiolucent lesion (35, 39). The current case series showed a tendency toward multilocular defect with soap-bubble or honeycomb appearance. Other complications like pathological fracture, paralyzation, and malocclusion are not very common. El Deeb et al. (11), Laskin et al. (40) and Shafer et al. (41) reported the presence of trauma history in this situation, which theoretically backs the assumption of intramedullary or subperiosteal hemorrhage.

In terms of diagnosing JABC, comprehensive pre-surgical examination is important, particularly the vascular type which accounts for 95% in JABCs. Brisk hemorrhage can be rebellious under this circumstance (5, 6, 8). Clinically, a multilocular, rapid-growing, destructive skeletal lesion is supposed to undertake digital subtraction angiography (DSA) or magnetic resonance angiography (MRA) to clarify the blood supplementary. Although the majority of oral and maxillofacial bone cysts cause no concern, thus hard tissue evaluation like panoramic radiograph and cone beam CT scan is generally sufficient. Highly vascularized lesions such as JABC and central hemangioma would result in unexpectable crisis, especially in the absence of blood preparation. Möller et al. (39) reported a JABC lesion furnished by ipsilateral external carotid artery. At the same time, Padwa et al. (17) found a displacement of internal maxillary superficial temporal arteries in a JABC patient. Wiatrak et al. (5) found an expansion of lingual, facial and internal maxillary arteries surrounding the pathological field. Related to rich blood supply, embolization before surgical procedure can be an effective measure to avoid uncontrollable bleeding.

Precisely because of the diversiform of JABC's clinical and radiographic features, the differential diagnosis ranges from simple bone cyst, central giant cell granuloma, ameloblastoma, odontogenic keratocyte, ossifying fibroma. Histopathology, as usual, is the golden standard for the final diagnosis. Surgical exploration can be an accessory for preliminary judgment.

Due to the absence of an epithelial wall, JABC was considered as a pseudocyst until 2004. It usually consists of a fibrous connective tissue stroma with blood-filled sinusoids, multinucleated giant cells, and irregular osteoid (42, 43). According to different histopathologic features, JABC can be segmented into 3 types. Vascular type is characterized by a loose stroma, numerous engorged blood-filled sinusoids. When occurring as the vascular type, it is very common to observe risky bleeding during surgery and extensive bony destruction with spread in the soft tissues. The solid type is identified by a dense, fibrous stroma, few blood vessels and without severe bleeding during the surgery (6, 44, 45). The third, mixed type lies somewhere in between the previous two variants. Proportion of each type varies between studies. Pelo et al. (6) reported that vascular type is the most common among the three classes. This concurs with Vergel et al. (46) who have reported that the solid variety was least common as opposed to Henriques et al. (47) who proposed that the solid variety is the most common.

When it comes back to the question of whether ABC is a primary or secondary lesion, opinions are controversial and still open to interpretation. Based on the classic etiologic hypothesis, primary ABC can result from any intramedullary or subperiosteal hemorrhage in which trauma may play a role in this process, or other altered hemodynamic state followed by the expansion of vascular bed and erosion of bone. Secondary ABC, on the other hand, is a phenomenon that originates from a previous osseous lesion (1, 3, 48). The exact proportion of primary and secondary ABC remains debatable as well. A 25% incidence of secondary JABCs with other pathological lesions was obtained in our study, similar to the findings of Pedwa et al. (22%) (17). This varies remarkably varies from Triantafillidou et al. research (50%) (1). Arora et al. (49) reported a result of 14.8% secondary ABC. Besides, Sun et al. reported an incidence of secondary JABCs of 76.5% with a detailed presentation of 17 JABC patients, which indicted that most secondary JABCs are lesions where the primary disease has been completely overlapped with aneurysmal change (50, 51). These differences may be a reflection of publication bias or reporting bias (where histopathology may not have been the focus of publication or the secondary lesion may be overlooked).

Another problem with JABC is the perplexing high recurrence. Ectopic anatomy and incomplete surgical procedure may both explain this issue in a specific type, that is, the exitance of communication between the primary bone lesion and surrounding arteries. Curettage is the most common and convenient solution and also holds a recurrence rate ranging from 21 to 50% in JABC patients (3). On this particular occasion, the supplementary artery is the fundamental problem rather than the cyst itself. Simple curettage or excision will not disconnect the blood supply nor correct the displacement of vessels which logically leads to a second expansion. Pedwa et al. (17), Trent et al. (13), Sarode et al. (32) and Gotmare et al. (33) all reported JABCs with initial curettage treatment then followed by recurrence and a second thorough hemi-mandibulectomy or maxillectomy were needed. Reddy et al. (25) also presented a relapse case in which this patient had taken over a second curettage along with cryotherapy. All of the above patients have not suffered any further recurrence, which verified that handling with blood supply and the primary bone lesion could be a meaningful treatment to avoid recurrence. Enlarged resection with ligation of nearby vessels, cryotherapy, embolization and sclerotherapy should be taken into consideration when countered with a recurrent JABC patient.

## Conclusion

From the discussion above, the acknowledgment of jaw aneurysmal bone cyst remains an ongoing challenge due to the epidemiology multiformity. The higher recurrence rate in secondary JABC may result from diverse histopathology of primary lesions, yet it requires further research. For clinical practitioners, the manifestation of JABC may be diverse, but in a single case, the biological behavior always matches pathological features, also prompting consistent and appropriate treatment. We here advocate comprehensive clinical and radiographic examination in suspected JABC patients, especially the transmission between the lesion and well-known arteriovenous in head and neck region, to avoid unexpected severe bleeding. Treatment should be cautiously considered in every patient to balance the recurrence and surgical injury.

## Author Contributions

YL: acquisition of data, laboratory or literature search. JZ: drafting of article and critical revision. JS: conception and design of review, final approval, and guarantor of manuscript. All authors contributed to the article and approved the submitted version.

## Conflict of Interest

The authors declare that the research was conducted in the absence of any commercial or financial relationships that could be construed as a potential conflict of interest.
