# Supplementary figures and images for: Clinicopathology and Recurrence Analysis of 44 Jaw Aneurysmal Bone Cyst Cases: A Literature Review
Source: Front Surg. 2021 Jun 23;8:678696. doi: 10.3389/fsurg.2021.678696 (PMC8260671; doi:10.3389/fsurg.2021.678696)

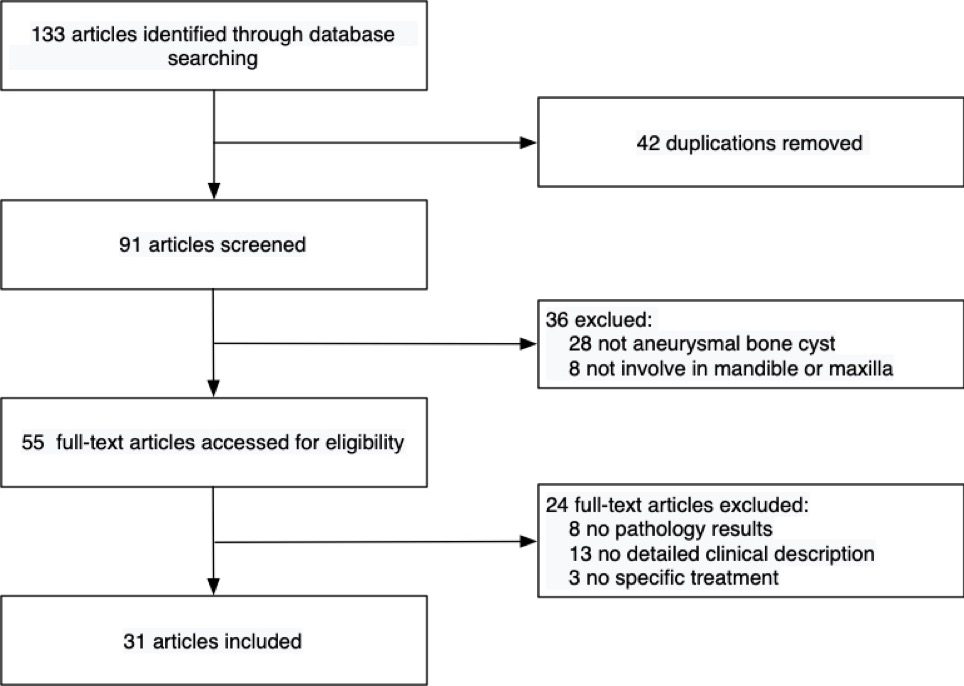

Supplement: Supplementary file 1 [file Image_1.JPEG]
